# Comment on “Perception of fertility, quality of life, and depression in women undergoing assisted reproductive treatment”

**DOI:** 10.5935/1518-0557.20250160

**Published:** 2025

**Authors:** Shubham Kumar

**Affiliations:** 1 Center for Global Health Research, Saveetha Medical College and Hospital, Saveetha Institute of Medical and Technical Sciences, Saveetha University, Chennai, India

Dear Editor,

We recently reviewed with great interest the article by Lourenço *et al*. (2025), titled “Perception of Fertility, Quality of Life, and Depression in Women Undergoing Assisted Reproductive Treatment. This cross-sectional study offers a compelling examination of the interplay between fertility-related quality of life (QoL), depression, and perceptions of infertility among 89 women undergoing assisted reproductive treatment at the Hospital de Clínicas de Porto Alegre. We commend the authors for their thoughtful use of validated tools such as the Fertility Quality of Life (FertiQoL) questionnaire, Fertility Problem Inventory (FPI), and Beck Depression Inventory (BDI), which provide a robust foundation for assessing the psychological dimensions of infertility. Their findings illuminate critical insights into the emotional burden faced by these women, and we appreciate their call for integrated psychosocial care in assisted reproduction programs an aspect of patient care that warrants greater attention.

The authors’ demonstration of a significant association between depression and reduced FertiQoL scores, particularly in the mind/body subscale (with a notable 13.4-point deficit among depressed patients), shows the profound impact of mental health on fertility-related QoL. We find their observation that social, conjugal/sexual, and parenthood-related FPI domains correlate strongly with FertiQoL scores especially valuable, as it highlights the multifaceted stressors these women encounter. The positive correlation between conjugal/sexual relationships and QoL, contrasted with the negative influence of social pressures and the need for parenthood, offers a nuanced perspective that enriches the literature. We applaud the authors for situating their findings within the broader context of existing research and Brazilian healthcare policies, such as the National Humanization Policy, which enhances the study’s relevance to clinical practice.

While the study’s strengths are evident, we respectfully suggest a few areas for further consideration that could amplify its impact. The cross-sectional design, though well-executed, limits causal inferences about the relationship between depression and QoL. A longitudinal approach, as the authors themselves note in their limitations, could provide deeper insights into how these factors evolve over the treatment trajectory an aspect we believe would be a natural extension of this work. Additionally, the finding that women with higher education reported lower QoL and higher depression scores is intriguing and somewhat counterintuitive to prior studies. We encourage the authors to explore this further, perhaps by examining potential mediators such as treatment expectations or socioeconomic pressures, which could clarify this unexpected trend.

Lastly, the exclusive focus on women, while justified given their disproportionate treatment burden, leaves room for a more comprehensive understanding by including male partners in future analyses. We believe this could align with the authors’ holistic approach and strengthen their advocacy for couple-centered psychosocial support.

Lourenço *et al*. (2025) have delivered a meticulously conducted study that meaningfully advances our understanding of the emotional landscape of infertility treatment. We sincerely praise their efforts and suggest these refinements to enhance the study’s already significant contributions. This work stands as a vital resource for clinicians and researchers alike, and we look forward to its influence on improving patient care in assisted reproduction
